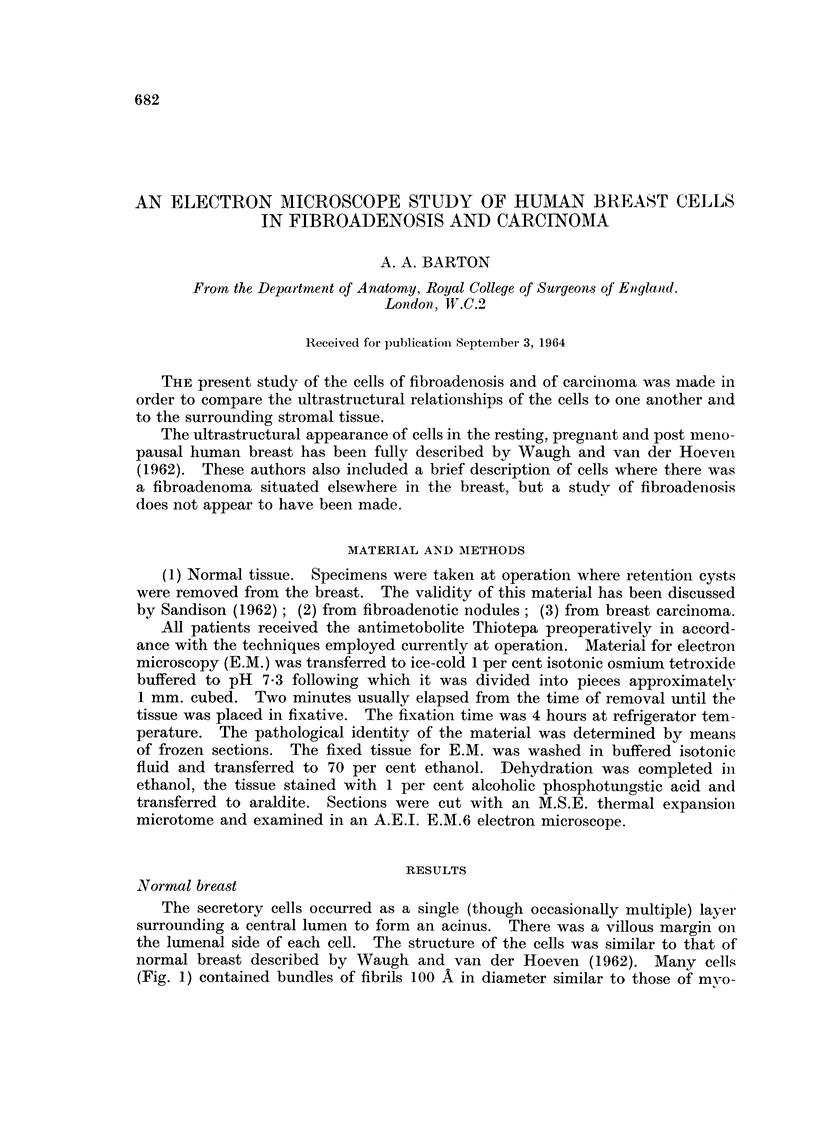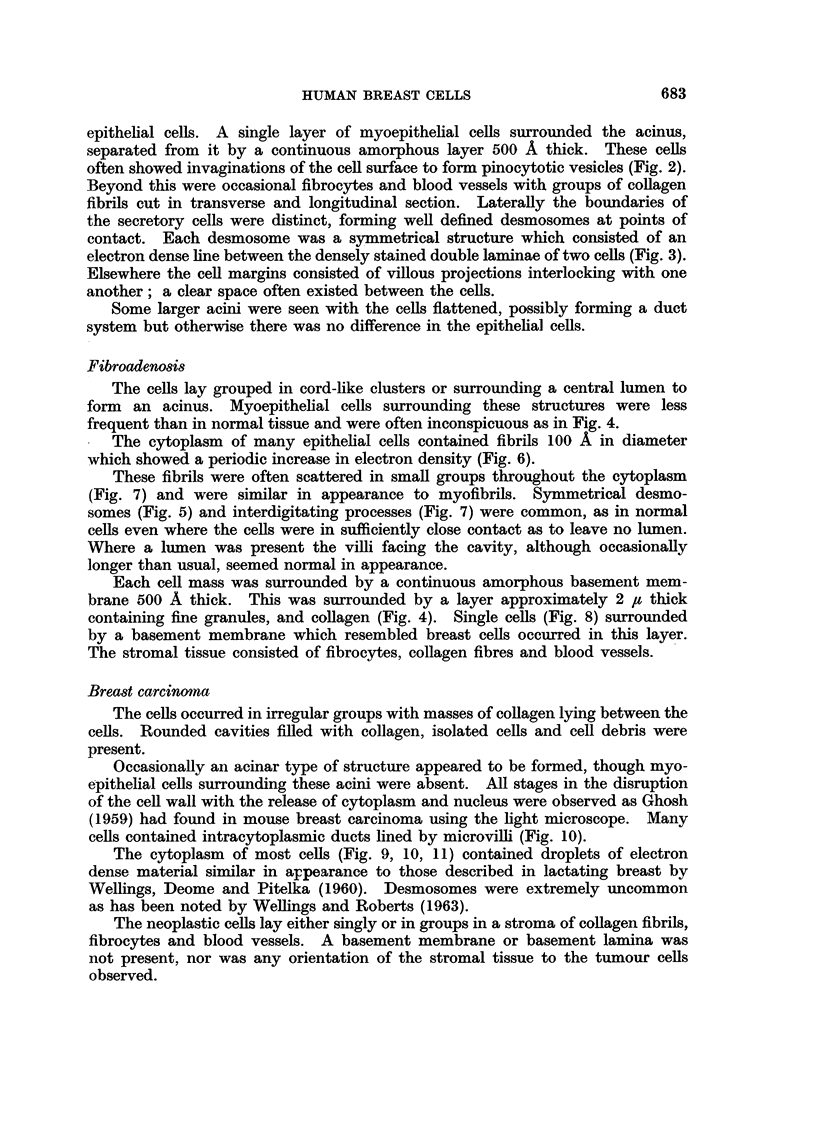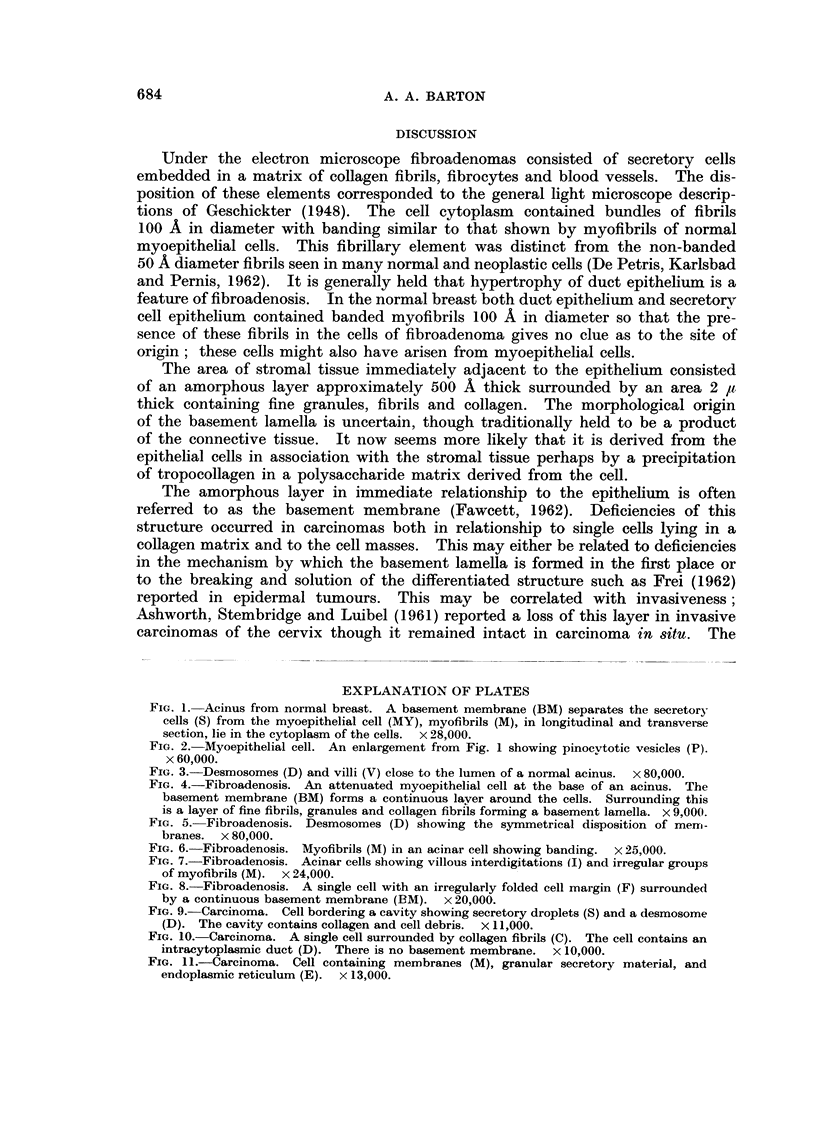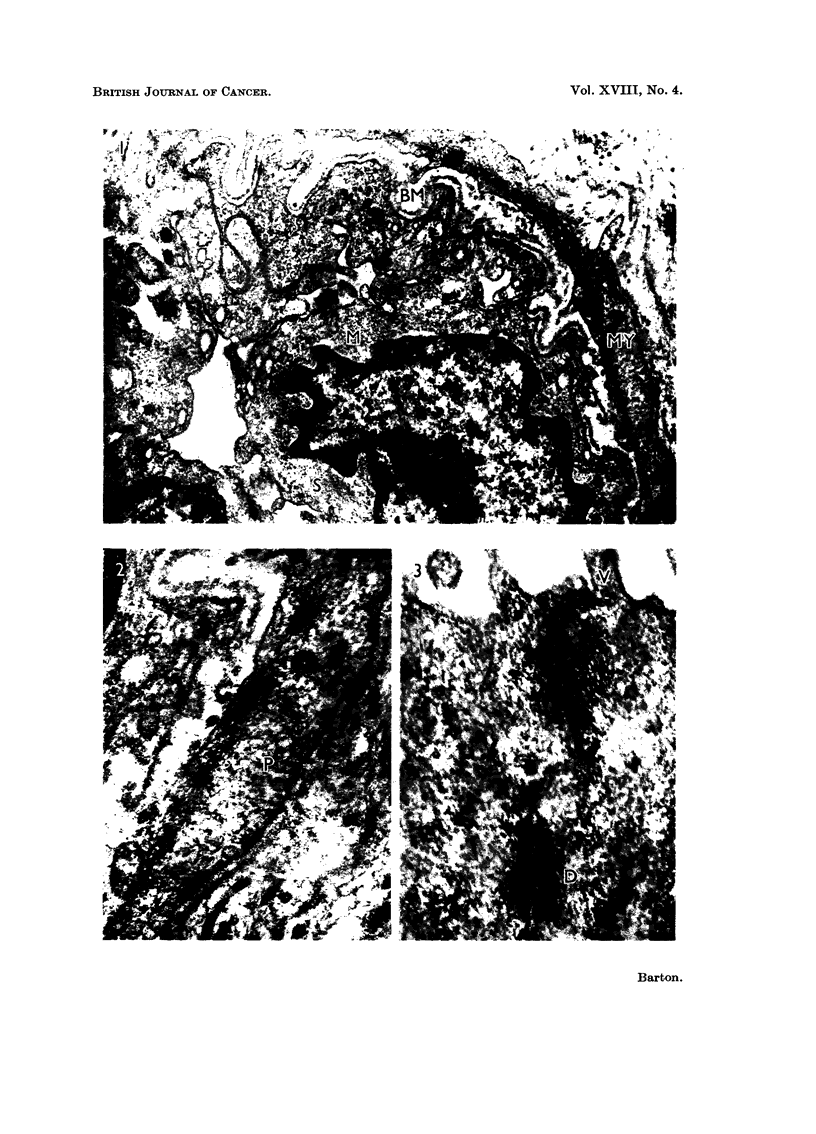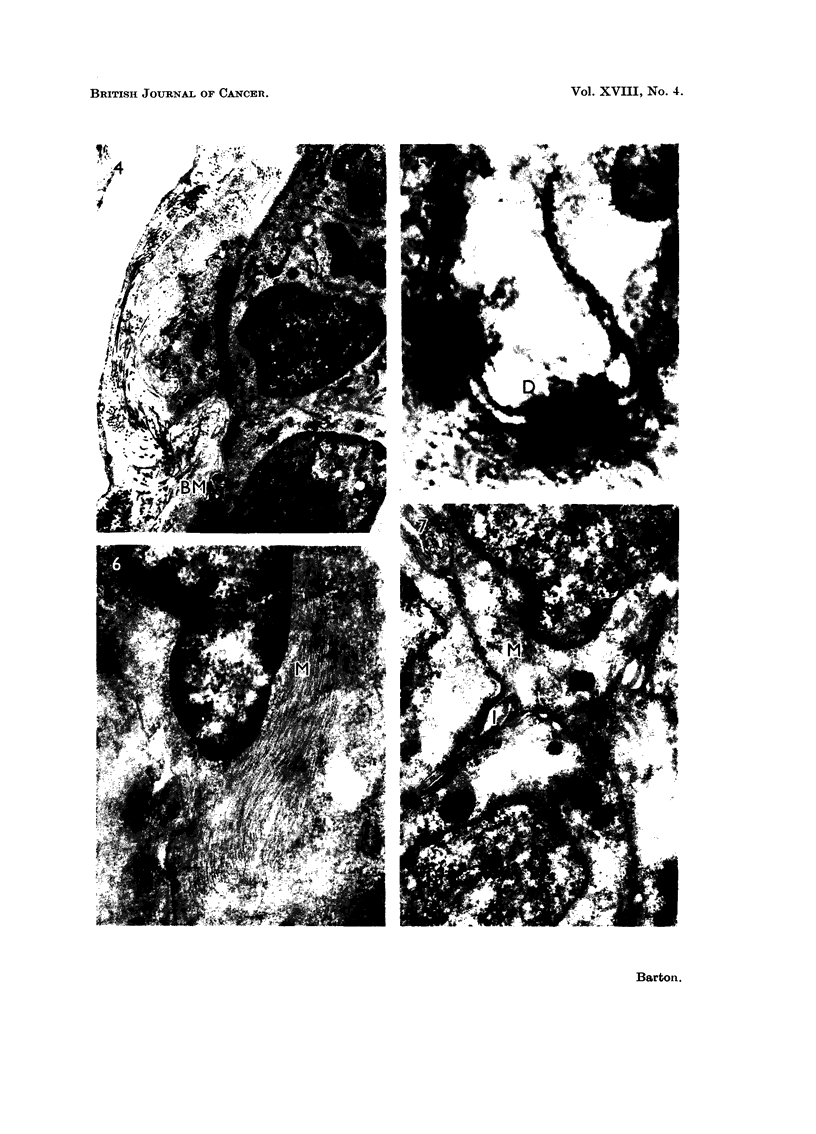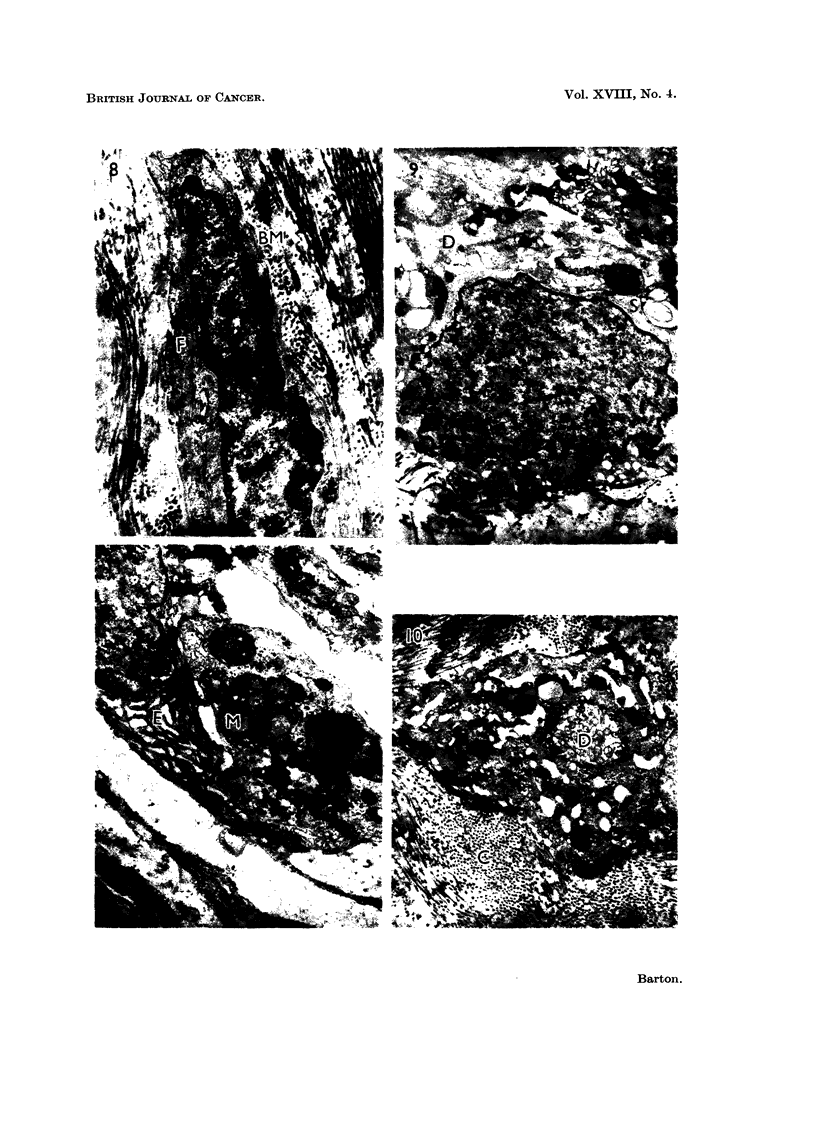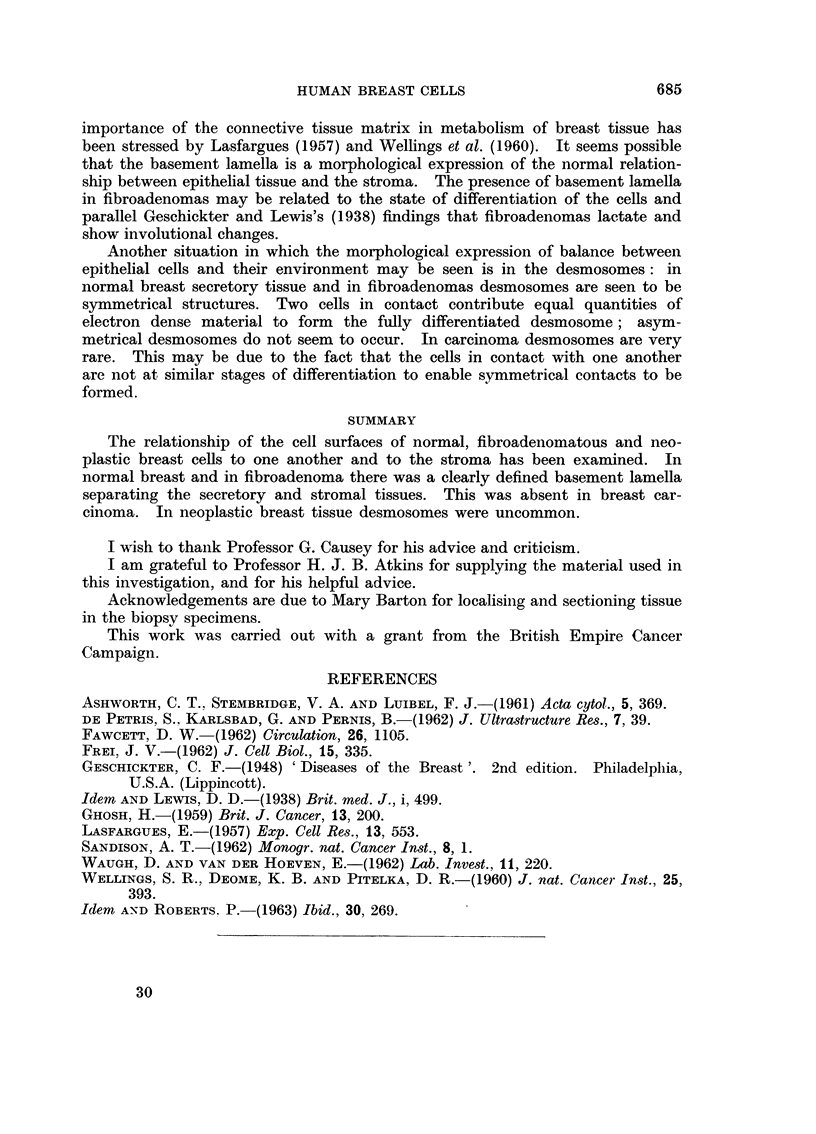# An Electron Microscope Study of Human Breast Cells in Fibroadenosis and Carcinoma

**DOI:** 10.1038/bjc.1964.78

**Published:** 1964-12

**Authors:** A. A. Barton

## Abstract

**Images:**


### AN ELECTRON MICROSCOPE STUDY OF HUMAN BREAST CELLS IN FIBROADENOSIS AND CARCINOMA

A. A. BARTON

*From the Department of Anatomy, Royal College of Surgeons of England, London, W.C.2*

Received for publication September 3, 1964

THE present study of the cells of fibroadenosis and of carcinoma was made in order to compare the ultrastructural relationships of the cells to one another and to the surrounding stromal tissue.

The ultrastructural appearance of cells in the resting, pregnant and post menopausal human breast has been fully described by Waugh and van der Hoeven (1962). These authors also included a brief description of cells where there was a fibroadenoma situated elsewhere in the breast, but a study of fibroadenosis does not appear to have been made.

### MATERIAL AND METHODS

(1) Normal tissue. Specimens were taken at operation where retention cysts were removed from the breast. The validity of this material has been discussed by Sandison (1962) ; (2) from fibroadenotic nodules ; (3) from breast carcinoma.

All patients received the antimetobolite Thiotepa preoperatively in accordance with the techniques employed currently at operation. Material for electron microscopy (E.M.) was transferred to ice-cold 1 per cent isotonic osmium tetroxide buffered to pH 7·3 following which it was divided into pieces approximately 1 mm. cubed. Two minutes usually elapsed from the time of removal until the tissue was placed in fixative. The fixation time was 4 hours at refrigerator temperature. The pathological identity of the material was determined by means of frozen sections. The fixed tissue for E.M. was washed in buffered isotonic fluid and transferred to 70 per cent ethanol. Dehydration was completed in ethanol, the tissue stained with 1 per cent alcoholic phosphotungstic acid and transferred to araldite. Sections were cut with an M.S.E. thermal expansion microtome and examined in an A.E.I. E.M.6 electron microscope.

### RESULTS

### *Normal breast*

The secretory cells occurred as a single (though occasionally multiple) layer surrounding a central lumen to form an acinus. There was a villous margin on the lumenal side of each cell. The structure of the cells was similar to that of normal breast described by Waugh and van der Hoeven (1962). Many cells (Fig. 1) contained bundles of fibrils 100 Å in diameter similar to those of myoepithelial cells. A single layer of myoepithelial cells surrounded the acinus, separated from it by a continuous amorphous layer 500 Å thick. These cells often showed invaginations of the cell surface to form pinocytotic vesicles (Fig. 2). Beyond this were occasional fibrocytes and blood vessels with groups of collagen fibrils cut in transverse and longitudinal section. Laterally the boundaries of the secretory cells were distinct, forming well defined desmosomes at points of contact. Each desmosome was a symmetrical structure which consisted of an electron dense line between the densely stained double laminae of two cells (Fig. 3). Elsewhere the cell margins consisted of villous projections interlocking with one another ; a clear space often existed between the cells.

Some larger acini were seen with the cells flattened, possibly forming a duct system but otherwise there was no difference in the epithelial cells.

*Fibroadenosis*

The cells lay grouped in cord-like clusters or surrounding a central lumen to form an acinus. Myoepithelial cells surrounding these structures were less frequent than in normal tissue and were often inconspicuous as in Fig. 4.

The cytoplasm of many epithelial cells contained fibrils 100 Å in diameter which showed a periodic increase in electron density (Fig. 6).

These fibrils were often scattered in small groups throughout the cytoplasm (Fig. 7) and were similar in appearance to myofibrils. Symmetrical desmosomes (Fig. 5) and interdigitating processes (Fig. 7) were common, as in normal cells even where the cells were in sufficiently close contact as to leave no lumen. Where a lumen was present the villi facing the cavity, although occasionally longer than usual, seemed normal in appearance.

Each cell mass was surrounded by a continuous amorphous basement membrane 500 Å thick. This was surrounded by a layer approximately 2 $\mu$ thick containing fine granules, and collagen (Fig. 4). Single cells (Fig. 8) surrounded by a basement membrane which resembled breast cells occurred in this layer. The stromal tissue consisted of fibrocytes, collagen fibres and blood vessels.

*Breast carcinoma*

The cells occurred in irregular groups with masses of collagen lying between the cells. Rounded cavities filled with collagen, isolated cells and cell debris were present.

Occasionally an acinar type of structure appeared to be formed, though myoepithelial cells surrounding these acini were absent. All stages in the disruption of the cell wall with the release of cytoplasm and nucleus were observed as Ghosh (1959) had found in mouse breast carcinoma using the light microscope. Many cells contained intracytoplasmic ducts lined by microvilli (Fig. 10).

The cytoplasm of most cells (Fig. 9, 10, 11) contained droplets of electron dense material similar in appearance to those described in lactating breast by Wellings, Deome and Pitelka (1960). Desmosomes were extremely uncommon as has been noted by Wellings and Roberts (1963).

The neoplastic cells lay either singly or in groups in a stroma of collagen fibrils, fibrocytes and blood vessels. A basement membrane or basement lamina was not present, nor was any orientation of the stromal tissue to the tumour cells observed.

### DISCUSSION

Under the electron microscope fibroadenomas consisted of secretory cells embedded in a matrix of collagen fibrils, fibrocytes and blood vessels. The disposition of these elements corresponded to the general light microscope descriptions of Geschickter (1948). The cell cytoplasm contained bundles of fibrils 100 Å in diameter with banding similar to that shown by myofibrils of normal myoepithelial cells. This fibrillary element was distinct from the non-banded 50 Å diameter fibrils seen in many normal and neoplastic cells (De Petris, Karlsbad and Pernis, 1962). It is generally held that hypertrophy of duct epithelium is a feature of fibroadenosis. In the normal breast both duct epithelium and secretory cell epithelium contained banded myofibrils 100 Å in diameter so that the presence of these fibrils in the cells of fibroadenoma gives no clue as to the site of origin ; these cells might also have arisen from myoepithelial cells.

The area of stromal tissue immediately adjacent to the epithelium consisted of an amorphous layer approximately 500 Å thick surrounded by an area 2 $\mu$ thick containing fine granules, fibrils and collagen. The morphological origin of the basement lamella is uncertain, though traditionally held to be a product of the connective tissue. It now seems more likely that it is derived from the epithelial cells in association with the stromal tissue perhaps by a precipitation of tropocollagen in a polysaccharide matrix derived from the cell.

The amorphous layer in immediate relationship to the epithelium is often referred to as the basement membrane (Fawcett, 1962). Deficiencies of this structure occurred in carcinomas both in relationship to single cells lying in a collagen matrix and to the cell masses. This may either be related to deficiencies in the mechanism by which the basement lamella is formed in the first place or to the breaking and solution of the differentiated structure such as Frei (1962) reported in epidermal tumours. This may be correlated with invasiveness ; Ashworth, Stembridge and Luibel (1961) reported a loss of this layer in invasive carcinomas of the cervix though it remained intact in carcinoma *in situ*. The

---

### EXPLANATION OF PLATES

FIG. 1.—Acinus from normal breast. A basement membrane (BM) separates the secretory cells (S) from the myoepithelial cell (MY), myofibrils (M), in longitudinal and transverse section, lie in the cytoplasm of the cells. ×28,000.

FIG. 2.—Myoepithelial cell. An enlargement from Fig. 1 showing pinocytotic vesicles (P). ×60,000.

FIG. 3.—Desmosomes (D) and villi (V) close to the lumen of a normal acinus. ×80,000.

FIG. 4.—Fibroadenosis. An attenuated myoepithelial cell at the base of an acinus. The basement membrane (BM) forms a continuous layer around the cells. Surrounding this is a layer of fine fibrils, granules and collagen fibrils forming a basement lamella. ×9,000.

FIG. 5.—Fibroadenosis. Desmosomes (D) showing the symmetrical disposition of membranes. ×80,000.

FIG. 6.—Fibroadenosis. Myofibrils (M) in an acinar cell showing banding. ×25,000.

FIG. 7.—Fibroadenosis. Acinar cells showing villous interdigitations (I) and irregular groups of myofibrils (M). ×24,000.

FIG. 8.—Fibroadenosis. A single cell with an irregularly folded cell margin (F) surrounded by a continuous basement membrane (BM). ×20,000.

FIG. 9.—Carcinoma. Cell bordering a cavity showing secretory droplets (S) and a desmosome (D). The cavity contains collagen and cell debris. ×11,000.

FIG. 10.—Carcinoma. A single cell surrounded by collagen fibrils (C). The cell contains an intracytoplasmic duct (D). There is no basement membrane. ×10,000.

FIG. 11.—Carcinoma. Cell containing membranes (M), granular secretory material, and endoplasmic reticulum (E). ×13,000.

Barton.

Barton.

8
BM
F

9
D
S

11
E
M

10
D
C

importance of the connective tissue matrix in metabolism of breast tissue has been stressed by Lasfargues (1957) and Wellings *et al.* (1960). It seems possible that the basement lamella is a morphological expression of the normal relationship between epithelial tissue and the stroma. The presence of basement lamella in fibroadenomas may be related to the state of differentiation of the cells and parallel Geschickter and Lewis's (1938) findings that fibroadenomas lactate and show involutional changes.

Another situation in which the morphological expression of balance between epithelial cells and their environment may be seen is in the desmosomes: in normal breast secretory tissue and in fibroadenomas desmosomes are seen to be symmetrical structures. Two cells in contact contribute equal quantities of electron dense material to form the fully differentiated desmosome; asymmetrical desmosomes do not seem to occur. In carcinoma desmosomes are very rare. This may be due to the fact that the cells in contact with one another are not at similar stages of differentiation to enable symmetrical contacts to be formed.

### SUMMARY

The relationship of the cell surfaces of normal, fibroadenomatous and neoplastic breast cells to one another and to the stroma has been examined. In normal breast and in fibroadenoma there was a clearly defined basement lamella separating the secretory and stromal tissues. This was absent in breast carcinoma. In neoplastic breast tissue desmosomes were uncommon.

I wish to thank Professor G. Causey for his advice and criticism.

I am grateful to Professor H. J. B. Atkins for supplying the material used in this investigation, and for his helpful advice.

Acknowledgements are due to Mary Barton for localising and sectioning tissue in the biopsy specimens.

This work was carried out with a grant from the British Empire Cancer Campaign.

### REFERENCES

Ashworth, C. T., Stembridge, V. A. and Luibel, F. J.—(1961) *Acta cytol.*, **5**, 369.

de Petris, S., Karlsbad, G. and Pernis, B.—(1962) *J. Ultrastructure Res.*, **7**, 39.

Fawcett, D. W.—(1962) *Circulation*, **26**, 1105.

Frei, J. V.—(1962) *J. Cell Biol.*, **15**, 335.

Geschickter, C. F.—(1948) 'Diseases of the Breast'. 2nd edition. Philadelphia, U.S.A. (Lippincott).

*Idem* and Lewis, D. D.—(1938) *Brit. med. J.*, i, 499.

Ghosh, H.—(1959) *Brit. J. Cancer*, **13**, 200.

Lasfargues, E.—(1957) *Exp. Cell Res.*, **13**, 553.

Sandison, A. T.—(1962) *Monogr. nat. Cancer Inst.*, **8**, 1.

Waugh, D. and van der Hoeven, E.—(1962) *Lab. Invest.*, **11**, 220.

Wellings, S. R., Deome, K. B. and Pitelka, D. R.—(1960) *J. nat. Cancer Inst.*, **25**, 393.

*Idem* and Roberts, P.—(1963) *Ibid.*, **30**, 269.